# Nucleotide composition of the Zika virus RNA genome and its codon usage

**DOI:** 10.1186/s12985-016-0551-1

**Published:** 2016-06-08

**Authors:** Formijn van Hemert, Ben Berkhout

**Affiliations:** Laboratory of Experimental Virology, Department of Medical Microbiology, Center for Infection and Immunity Amsterdam (CINIMA), Academic Medical Center, University of Amsterdam, Meibergdreef 15, 1105 AZ Amsterdam, The Netherlands

**Keywords:** Zika virus, ZIKV, Viral RNA genome, Nucleotide composition, Nucleotide bias, Purine-rich, Codon usage, RNA structure, Flavivirus

## Abstract

**Background:**

RNA viruses have genomes with a distinct nucleotide composition and codon usage. We present the global characteristics of the RNA genome of Zika virus (ZIKV), an emerging pathogen within the Flavivirus genus. ZIKV was first isolated in 1947 in Uganda, caused a widespread epidemic in South and Central America and the Caribbean in 2015 and has recently been associated with microcephaly in newborns.

**Methods:**

The nearly 11 kb positive-stranded RNA genome of ZIKV was analyzed for its nucleotide composition, also in the context of the folded RNA molecule. Nucleotide trends were investigated along the genome length by skew analyses and we analyzed the codons used for translation of the ZIKV proteins.

**Results:**

ZIKV RNA has a biased nucleotide composition in being purine-rich and pyrimidine-poor. This preference for purines is a general characteristic of the mosquito-borne and tick-borne flaviviruses. The virus-specific nucleotide bias is further enriched in the unpaired, single-stranded regions of the structured ZIKV RNA genome, thus further imposing this ZIKV-specific signature. The codons used for translation of the ZIKV proteins is also unusual, but we show that it is the underlying bias in nucleotide composition of the viral RNA that largely dictates these codon preferences.

**Conclusions:**

The ZIKV RNA genome has a biased nucleotide composition that dictates the codon usage of this flavivirus. We discuss the evolutionary scenarios and molecular mechanisms that may be responsible for these distinctive ZIKV RNA genome features.

## Background

Several members of the Flavivirus genus are the causative agents of significant diseases in humans, livestock and wildlife. These include dengue virus that affects more than 50 million people worldwide each year, West Nile virus and Japanese encephalitis virus that caused outbreaks in North America and Asia, respectively [1]. Zika virus (ZIKV) is an emerging pathogen in the family *Flaviviridae* that was first isolated in 1947 from a sentinel rhesus monkey placed in the Zika Forest near Lake Victoria in Uganda [2]. ZIKV is transmitted by mosquitoes, especially *Aedes africanus*, but the virus was also isolated from other *Aedes* species (reviewed in [3]). ZIKV infections of humans was first described in 1964 [4], causing a febrile illness with dengue fever like symptoms [5]. Sporadic cases were reported in sub-Saharan Africa and Southeast Asia, followed by an outbreak in Micronesia in 2007 and major epidemics that started around 2013 in New Caledonia, the Cook Islands, French Polynesia and Easter Island [6, 7]. A rather dramatic increase in the number of ZIKV cases was reported from the Americas starting in 2015, Brazil being the most affected country with around 1 million cases reported at the end of 2015 [8–10]. Here, also cases of neurological manifestations and the Guillain-Barré syndrome were described. Recent reports indicate a significant increase in the number of microcephaly cases among newborns in northeast Brazil, suggesting that ZIKV infection in pregnancy may trigger fetal malformations [11]. Neural progenitor cells can be infected by this virus, leading to attenuation of their growth [12].

Given the clinical relevance, we performed a detailed analysis of several features of the ZIKV RNA genome, including the nucleotide (nt) composition, also in the context of the structured RNA genome, and the viral codon usage. This insight can be central to the understanding of factors that govern virus evolution. Mutation pressure has been shown to be the dominant factor shaping the nucleotide composition and codon usage in mammalian genomes [13–15]. The ZIKV genome of almost 11,000 nts encodes a single polyprotein of 3419 amino acids that is cleaved by the viral serine and cellular furin proteases into the functional domains: the Capsid (C), Precursor of membrane (prM), Envelope (E) and 7 non-structural proteins (NS) [2]. We report that the nucleotide composition of the ZIKV virus genome is strongly biased and this bias directly influences the codons used for translation of the viral proteins.

## Methods

### ZIKV sequences

Viral RNA genome sequences were obtained from GenBank. The MR-766 prototype ZIKV strain originates from the index case: a monkey infected in 1947 in Uganda (Genbank entry NC_012532). Other ZIKV isolates used: KU497555 (Brazil), KU509998 (Haiti), KU501215 (Puerto Rico), KU312312 (Suriname), KU647676 (Martinique), KJ776791 (French Polynesia), KU701217 (Guatamala), KU681082 (Philippines) and KF268950 (Central African Republic). The full genome sequences were manually curated into *bona fide* open reading frames.

### Maximum Likelihood (ML) phylogenetic analysis

Phylogenetic and molecular evolutionary analyses were conducted with MEGA v6 [16]. The open reading frames (ORFs) of the different ZIKV strains were translated into amino acid sequences, which were aligned by means of the MUSCLE tool. The JTT + G model for assessing amino acid replacements during ZIKV evolution turned out to be the best fitting model judged by BIC score (Bayesian Information Criterion, 22469.52863) and AICc value (corrected Akaike Information Criterion, 22317.61634). Non-uniformity of evolutionary rates among sites was modeled by a discrete Gamma distribution (+G = 0.554328239) with 5 rate categories. The ML value for model selection was logL = −11140.79817. All sites were used for phylogenetic analysis. A bootstrap test (1000 replicates) indicated robustness of the analysis. The hypothetical ancestral sequence (MRCA, Most Recent Common Ancestor) was constructed by means of the FastML server with the advanced options activated [17]. The MLtree was rooted on the MRCA branch to show the evolutionary course of events.

### RNA structure prediction

RNA secondary structure prediction was performed by the MFold v3.6 algorithm with default settings [18]. The MFold output file provided the ss-count, a frequency value that indicates whether an individual nucleotide (nt) is unpaired in the collection of folded structures (maximally 50). We scored an unpaired nt (single-stranded, "ss") if present in at least half of the RNA structures. Nts with a lower ss-count were scored double-stranded ("ds"). Excel was used for ss/ds discrimination and we generated fasta files to determine the nucleotide composition in MEGA v6 [16]. Because the size limit for submission to the MFold server is 9000 nts, the ZIKV RNA genome was partitioned into two fragments with 1000 nts overlap. We arithmetically averaged the ss-count data in the overlap before ss/ds discrimination was performed.

### Skew analysis

Base composition along the complete RNA genome length and the accompanying ss and ds fasta files was analyzed by cumulative skew diagrams using overlapping windows [19, 20]. Overlapping windows were defined around 1 % of the sequence length with a step size of 20 % of the window size, which generated about 500 data points per analysis irrespective of sequence length. A skew between nts N1 and N2 is defined as (N1 − N2)/(N1 + N2). A positive value indicates that N1 exceeds N2.

### Codon usage

The single ZIKV reading frame was analyzed using the “Nc-plot”, which plots the effective number of codons (ENC-values) versus the GC-content at the 3^rd^ codon position (GC3) [21]. A continuous line indicates ENC values expected (ENCexp) for random codon usage at that particular GC3 value. Deviation from this line in the direction of lower ENC-values (observed ENC values, ENCobs) points to the selection of a preferred set of codons as described for highly expressed genes in yeast [22] and *Escherichia coli* [23]. The ratio ENCobs/ENCexp provides an easy measure of this deviation. A ratio value of 1 indicates zero codon bias. Values close to 1 (0.8 to 1.0) indicate very weak or virtually absent codon bias. ENC and GC3 values of sequence data were determined by means of Simmonic 2005 v1.5 software [24]. ENC and GC3 values human and *Aedes* genes were derived from codon usage tables [25]. All calculations were performed in Excel v14.0.7128.5000.

## Results

### Nucleotide composition of the ZIKV RNA

We first analyzed the complete RNA genome of the ZIKV MR-766 prototype strain [2], which originates from a sentinel monkey that became infected in Uganda in 1947 (Genbank entry NC_012532). This positive-stranded RNA genome contains a single extended open reading frame (ORF) that encodes the viral polyprotein, which is subsequently processed into the different structural and enzymatic components. The ZIKV RNA genome is 10794 nucleotides (nts) long, with a short 5’-untranslated region (UTR) of 106 nts and a 3’-UTR of 428 nts. The four possible nucleotides are not used at equal frequencies, the genome composition ranges from 2305 U (21.3 %) to 3139 G (29.1 %). Table 1 summarizes these numbers and provides some further details. Overall, the RNA genome is enriched for purines (G + A, 56.8 %) over pyrimidines (U + C, 43.2 %), yielding a Pu/Py ratio of 1.31. We will relate these ZIKV properties to that of other flaviviruses in the discussion.

**Table 1:** ZIKV RNA composition

ZIKV RNA	nts	A	U	C	G	Pu/Py
complete	10794	2991 (27.7 %)	2305 (21.3 %)	2359 (21.9 %)	3139 (29.1 %)	1.31
5’UTR	106	29	31	16	30	
3’UTR	428	113	72	118	125	
5’ + 3’UTR	534	142 (26.6 %)	103 (19.3 %)	134 (25.1 %)	155 (29.0 %)	1.25

**Fig. 1:**
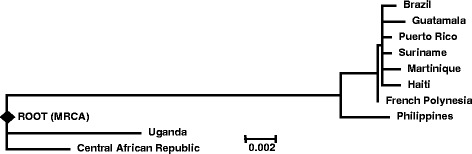
Molecular phylogeny of ZIKV strains. The polyprotein ORFs were translated into the amino acid sequence and a hypothetical ancestral sequence, generated with FastML, was added to the alignment. The Most Recent Common Ancestor (MRCA) was predicted to have been around 91 years ago, see the results section for further details. We performed 1000 bootstrap replicates that support the robustness of the phylogenetic analysis. The Log Likelihood value (LnL) of tree building amounted to −11055.85. The scale bar indicates the number of amino acid substitutions per site

These basic characteristics do not deviate significantly for other ZIKV isolates. We analyzed two early isolates (Uganda and Central African Republic) and 8 recent isolates. Phylogeny of these 10 strains is presented in Fig. 1, which is in agreement with published data [11, 26, 27]. ZIKV from the Philippines is ancestral to those constituting the recent epidemic in southern America. The root of the trifurcated tree indicates that a putative most recent common ancestor (MRCA) circulated in Africa approximately 91 years ago, assuming a constant virus evolution rate and an age of 68 years for the prototype Uganda virus. The typical nucleotide composition and purine preference are common features among the ancient and more recent taxa of this ZIKV collection. For instance, the recently isolated Natal RGN strain isolated from a Brazilian child with microcephaly [11] contains 21.4 % U and 29.2 % G, with a preference for purines of 56.6 %. We conclude that these nt characteristics are constant properties among ZIKV variants, at least those circulating in the last century.

This nt bias is likely to influence the codon usage in the single ORF that encodes the ZIKV polyprotein, but in fact the same nt trends are apparent for segments of the RNA genome that are not translated into protein: the short 5’-untranslated region (5’UTR) and 3’UTR of the ZIKV genome (Table 1). These regions are very short, 106 and 428 nts respectively for the MR-766 prototype genome, making a statistically sound analysis of the nt-count precarious, but when combined these domains exhibit exactly the same nt ranking order as the full-length RNA: G (29.0 %) > A (26.6 %) > C (25.1 %) > U (19.3 %). Local fluctuations may occur especially near the 5’ and 3’ termini of the genome due to the presence of essential sequence elements that are involved in viral genome replication. For instance, the U-count of the 5’UTR is elevated, in part due to the presence of four U3 stretches in this 106-nt segment. Conservation of these specific molecular signals among different ZIKV strains argues for such a biological role, e.g. the 107-nts 5’UTR of the Natal RGN isolate encodes three U3 and two U4 stretches. In addition, the genome ends may encode specific RNA structures with a replicative function [2].

### Composition of the structured RNA genome

We previously reported that the unequal nucleotide composition of a viral RNA genome may become even more biased in unpaired domains of the folded RNA molecule. This initial study was performed for HIV-1 RNA, using both MFold-generated structure prediction and the experimentally probed RNA structure [28, 29]. The structure of the ZIKV RNA genome was predicted by MFold. We investigated maximally 50 folded structures to provide each individual nt a frequency value of being unpaired (single-stranded, ss) in this collection of predicted structures. We subsequently counted the number of ss and ds (double-stranded, paired) positions (Table 2). Paired positions are in excess over unpaired nucleotides in these folded RNA genomes, with a dsRNA value of 62.0 %. One major and several minor trends can be recognized. First and most importantly, we observed a strong A-accumulation for the unpaired ss segments of the ZIKV RNA genome (from 27.7 to 44.7 %), which equally means A-depletion (from 27.7 to 17.2 %) in the paired ds genome domains. All non-A nts seem to compensate for these strong A-movements. In other words, G, U and C increase their prevalence in the paired domains, but lose incidence in the unpaired domains due to the prominent A-pressure. The unpaired part of ZIKV RNA is particularly A-rich (44.7 %) and C-poor (14.6 %), with a multitude of purines (G + A, 68.3 %). These properties may be relevant for interaction with intracellular sensors of the innate immune systems in humans and *Aedes* [30–32]. A more definitive analyses should be performed when the experimentally probed structure of the ZIKV virus RNA genome becomes available.

**Table 2:** Nucleotide composition in ss/ds segments

ZIKV RNA	nts	A	U	C	G
all	10794	2991 (27.7 %)	2305 (21.3 %)	2359 (21.9 %)	3139 (29.1 %)
ds	6687	1154 (17.2 %)	1604 (24.0 %)	1758 (26.3 %)	2171 (32.5 %)
ss	4107	1837 (44.7 %)	701 (17.1 %)	601 (14.6 %)	968 (23.6 %)

### Genome skew analysis

**Fig. 2:**
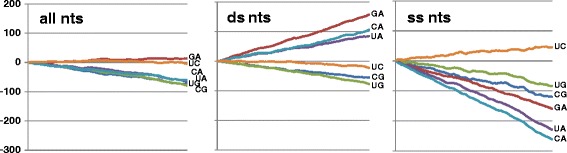
Nucleotide skew analysis of the ZIKV RNA genome. Skew values (N1 − N2)/(N1 + N2) were calculated in overlapping windows along the sequence. Window size was set at 1 % of the length of the sequence with a step size of 20 % of the window size, resulting in approximately 500 data points comprising the X-axis. We used the same Y-axis for the cumulative skew values to allow a direct comparison of the compositional signatures of different genome parts: “all nts”, “ds nts” and “ss nts”

A powerful way to visualize trends in nt usage is a nucleotide skew analysis along the viral genome (Fig. 2). The general trends presented above were confirmed. Note that GA in skew language does not represent a base pair, but rather a comparison of the number of G with the number of A nts. The skew lines are generally straight, indicating that the observed trends are steady along the 11 kb genome, without significant positional effects along the viral genome, e.g. in the 5’ and 3’UTRs versus the extended ORF. The skew analysis of the complete genome is presented in the left panel (all nts). This assessment underscores the preference of purines over pyrimidines as any direct Py-Pu comparison is won by the latter, resulting in a declining line for CA, UA, CG and UG. Only the Pu-Pu (GA) and Py-Py (UC) comparisons showed a lack of favorite nt.

We subsequently analyzed the ds and ss positions separately (Fig. 2, ds in middle panel, ss in right panel). The skew lines show more divergence in both the ds and ss segments compared with the all-nt skew analysis. It is also immediately apparent that the ds and ss positions act as communicating vessels. The gain of the A-count in the ss segment (strongly declining lines for GA, UA and especially CA) is mirrored by a loss in the ds compartment (strongly rising line for UA, CA and GA). G seems the second best option in the ss compartment (declining line for UG and CG). In fact, these trends do not mimic any of the virus-specific trends that we described previously, e.g. for retroviruses and coronaviruses [20, 33–35].

### Codon analysis

It is quite usual to perform an autonomous codon analysis, but we purposely first presented the general genome characteristics as these may largely influence the codon usage. It is immediately clear that ZIKV does not use all codons at equal frequency, but the patterns seem to vary (Table 3). Among the 4-codon groups (Ala, Gly, Pro, Thr and Val), but also the 4-codon set within the 6-codon groups (Leu, Arg and Ser), two quite opposite patterns can be recognized. Either the A-ending codons prevail (Pro, Ser, Thr), or the G-ending codons win (Leu, Val). Some outliers are also apparent: the Gly 4-codon set prefers both A and G, the Ala 4-codon set prefers C with A as second best, and the 4-codon set within the 6-codon group Arg has C as the best option, but with G as the second best. The opposing A/G trend is even more remarkable. When A wins, G does not become the second-best, but always drops to the lowest value. Inspection of the codons provides a possible explanation for this restriction as the suppressed codons encode a CpG motif (marked in bold and by underlining in Table 3). The CpG motif is a methylation signal in DNA, but is also recognized by host innate immune sensors as a pathogen signature [36], and is discriminated against in many RNA viruses, including poliovirus and the *Flaviviridae* [37, 38]. For ZIKV RNA, a striking GpC/CpG ratio of 2.17 was scored (analysis not shown). In other words, it seems that G wins unless a CpG motif is created.

**Table 3:** ZIKV codon usage

AA	Codon^a^	Number	Proportion	4-group^a^	6-group^1a^	G/A	U/C	|	AA	Codon^a^	Number	Proportion	4-group^a^	6-group^a^	G/A	U/C
Ala	G**CG**	30	0.11	C > A > U > **G**				|	Asn	AAU	36	0.35				C > U
	GCA	80	0.28					|		AAC	68	0.65				
	GCU	77	0.27					|	Pro	C**CG**	11	0.08	A > C > U > **G**			
	GCC	96	0.34					|		CCA	65	0.46				
Cys	UGU	35	0.55				U > C	|		CCU	28	0.2				
	UGC	29	0.45					|		CCC	38	0.27				
Asp	GAU	61	0.4				C > U	|	Gln	CAG	38	0.5			G = A	
	GAC	93	0.6					|		CAA	38	0.5				
Glu	GAG	115	0.52			G > A		|	Arg	AGG	57	0.26		A > G		
	GAA	107	0.48					|		AGA	93	0.42				
Phe	UUU	45	0.49				C = U	|		CGG	21	0.1		C > G > U > A		
	UUC	46	0.51					|		CGA	11	0.05				
Gly	GGG	67	0.22	A > G > C > U				|		CGU	14	0.06				
	GGA	150	0.49					|		CGC	24	0.11				
	GGT	40	0.13					|	Ser	AGU	36	0.18		C = U		
	GGC	51	0.17					|		AGC	38	0.19				
His	CAU	34	0.46				C > U	|		U**CG**	18	0.09		A > U > C > **G**		
	CAC	40	0.54					|		UCA	50	0.25				
Ile	A**UA**	51	0.29					|		UCU	29	0.15				
	AUU	46	0.26					|		UCC	28	0.14				
	AUC	78	0.45					|	Thr	A**CG**	20	0.09	A > C > U > **G**			
Lys	AAG	111	0.59			G > A		|		ACA	91	0.4				
	AAA	77	0.41					|		ACU	53	0.23				
Leu	UUG	69	0.22		G > **A**			|		ACC	62	0.27				
	U**UA**	20	0.06					|	Val	GUG	115	0.42	G > C > U > **A**			
	CUG	94	0.3		G > C > U > **A**			|		G**UA**	31	0.11				
	C**UA**	31	0.1					|		GUU	55	0.2				
	CUU	46	0.15					|		GUC	70	0.26				
	CUC	53	0.17					|	Trp	UGG	92	1				
Met	AUG	130	1					|	Tyr	UAU	38	0.44				C > U
								|		UAC	49	0.56				

^a^CpG and UpA in bold/underlined

A second dinucleotide motif that is discriminated against is UpA with an ApU/UpA ratio of 1.86. This explains most cases where A-ending codons are losing (marked in bold and by underling in Table 3), e.g. within the Leu 6-codon set it clarifies the choices made for both the 2-codon and 4-codon sets. The complete dinucleotide analysis (not shown) indicates that the all purine (GpA/ApG) and all pyrimidine (CpU/UpC) choices are unbiased compared to the four purine/pyrimidine combinations (results not shown). Both CpG and UpA discrimination have been reported for other flaviviruses [39, 40], but surprisingly little attention was given to the purine/pyrimidine composition as the potential unifying signature.

Overall, the purines G or A seem to dominate the codon choices made, and U/C choices seem relatively balanced. The G-bias is also apparent in the 2-codon groups (G/A column in Table 3), where G wins over A in 3 of 5 case, with a draw for the Gln group and a unique A-win for the 2-codon set within the outlier Arg 6-codon group. Only modest effects were scored for the U/C choice in the 2-codon groups, but C wins in 4 out of 7 cases with two draws, consistent with the overall nt count. Thus, codon usage in ZIKV RNA seems to follow the nt compositional trend with regard to the purine/pyrimidine bias that is present across the viral genome.

**Fig. 3:**
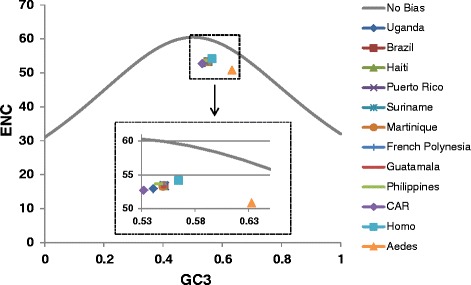
Codon ENC analysis of ZIKV RNA. The effective number of codons (ENC-values, Y-axis) of the single ZIKV ORF (3420 codons) was plotted against the GC-content at the 3rd synonymous codon positions (GC3-values, X-axis). The continuous grey line represents theoretical ENC values for random codon usage as a function of GC3. Deviation from this line in the direction of lower ENC-values points to selection of a preferred set of codons. We analyzed codon usage for several ZIKV isolates and the two host species (homo sapiens and *Aedes aegypti*). The latter two values are based on the following numbers of codons (with the number of genes in parentheses, see [25]): human: 40,662,582 (93,487) and *Aedes aegypti*: 257,935 (585)

Codon usage in the single ZIKV ORF was also analyzed by means of an “Nc-plot” (Fig. 3), with the effective number of codons (ENC-values) plotted versus the GC-content at the 3^rd^ codon positions (GC3). The continuous grey line indicates the ENC values expected (ENCexp) for random codon usage at a particular GC3 value. Deviation from this line in the direction of lower ENC-values (observed ENC values, ENCobs) points to the selection of a preferred set of codons. The ratio ENCobs/ENCexp provides an easy measure for the extent of deviation: the value of 1 (ENCobs = ENCexp) indicates the absence of any codon bias, values below 0.8 may suggest weak codon bias, and even lower values can advocate stronger codon bias. We included two early and several recent ZIKV isolates, but all cluster in a very tiny area of the Nc-plot, confirming their very close genetic relationship. Most importantly, the ENCobs/ENCexp ratio of all ZIKV isolates is above 0.88, which is close to the unbiased value. We also plotted ENC/GC3 values derived from average codon usage in a set of human and mosquito mRNAs (Homo and *Aedes* in Fig. 3). ZIKV and human ENC/GC3 values are very similar. The mean GC3 value for *Aedes* genes is somewhat higher (0.63) compared with the values for ZKIV and human genes (0.53). Between virus and hosts we did not observe different codon preferences, which are related to codon bias by translational control and indicated by low ENCobs/ENCexp values. Overall, this analysis suggests that the virus-specific codon usage trends reflect the biased nt composition of these viral RNA genomes.

## Discussion

This survey of the ZIKV RNA genome indicates a preference for purines over pyrimidines that is enhanced in the unpaired domains of the viral RNA and that influences the codons used for translation of the viral proteins. No significant differences were found among the highly related ZIKV strains concerning these basic properties, ranging from the prototype Uganda strain isolated in 1947 to recent south American isolates. The strong correlation between nucleotide composition and codon usage bias suggests that mutation pressure in ZIKV is an important determinant of the codon bias observed. This finding is consistent with previous findings for other viruses, which demonstrate a wide variation in codon usage that usually correlates with the viral RNA-specific nucleotide composition [41]. The ZIKV nt characteristics are quite distinctive from those of other viruses and may thus help to comprehend virus evolution and to provide an additional tool for virus classification purposes or the development of diagnostic reagents for improved surveillance of this class of emerging pathogens. A quick comparison of ZIKV to other members of the family *Flaviviridae*, including the major pathogens dengue virus [42], yellow fever virus, Japanese encephalitis virus, West Nile virus [43] and hepatitis C virus, indicated a similar purine-preference for the mosquito-borne and tick-borne flaviviruses and the pestivirus genus, but not for the hepacivirus group (results not shown). Intriguingly, these purine-loving viruses seem to favor either A or G, similar to the “communicating” pyrimidine vessels described for coronaviruses [35]. Although quite extensive codon analyses have been conducted for flaviviruses [42–50], as far as we know the typical purine/pyrimidine pattern has not yet been described previously.

We previously presented two possible causes for the presence of viral RNA genomes with a biased nt-composition [51]. One frequently entertained possibility is that this is due – over evolutionary times - to mutational bias of the viral polymerase [52]. Alternatively, we suggested a specific genome composition may be selected for a specific function, e.g. to facilitate RNA packaging in the virion particle or to prevent recognition by innate immune sensors in the infected cell. For HIV-1, there are recent indications to support both functional scenario’s [30, 53]. For ZIKV, one cannot formally exclude that functions are executed by the viral minus-RNA strand, the critical replication intermediate that in terms of nt-composition is the mirror image of the viral plus-strand RNA genome. In addition, the double-stranded RNA replication intermediate and its nt-composition and structure may be screened by several innate immune sensors in the human, monkey and *Aedes* hosts.

We previously reported that virus-specific compositional signatures are commonly enhanced in the unpaired domains of a structured RNA genome. This is true for HIV-1, with an average A-count of 36.2 % that increases to 47.5 % in the unpaired genome segments, and also for other retroviruses with a distinct nt bias [28, 34]. We recently analyzed coronaviruses and reported some common characteristics (high U, low C count), but also species-specific signatures that differentiate the pathogenic MERS/SARS strains from other coronaviruses [35]. Again, nucleotide biases were boosted in the unpaired domains of the viral RNA genome. The concentration of nt-bias in certain genome domains may have been selected for a certain function.

Many reports dwell on the exotic codon usage employed by viruses. For instance, a recent report documented codon usage adaptation in pandemic ZIKV virus strains [54]. However, a sobering finding is that this bias usually coincides with a bias in nt-composition of the viral RNA genome, and therefore does not represent translational selection of certain codons and/or the matching tRNA species. This also seems true for ZIKV, which preferentially uses A- or G-rich codons, but this seems to be a direct consequence of having a purine-rich RNA genome (56.8 %). HIV is the only virus in this collection that combines A-accumulation with weak translational selection by means of codon bias. Previously, we have even documented a tendency in HIV proteins for selection of amino acids encoded by A-rich codons [55]. Codon usage can become an important aspect when it comes to optimization of protein production, e.g. in the context of ZIKV vaccine development where efficient viral gene expression may be required to generate immunity [56]. Given the biased codon usage of this virus, it is important to change towards synonymous codons that are more favored in the relevant production platform, e.g. human cell lines.
